# Goal setting for thriving and sustainable success in the performing arts: taking inspiration from nature

**DOI:** 10.3389/fpsyg.2026.1738842

**Published:** 2026-04-01

**Authors:** Veronika J. Lubert, Lilian Peters

**Affiliations:** 1 School of Music, Lucerne University of Applied Sciences and Arts, Lucerne, Switzerland; 2 Independent Researcher, Munich, Germany

**Keywords:** acting, dance, goal setting, music, qualitative analysis, SMART goals

## Abstract

Goal setting has been studied extensively across domains such as sports, business, and education, but rarely in the performing arts. In this perspective paper, we explore how nature and its synergistic processes can serve as a metaphorical heuristic to inspire goal setting in acting, dance, and music. We do so by drawing on studies of ecosystems and recent literature reviews, discussing different goal-setting approaches, and providing insights from a qualitative case study with performing artists. Goals may enhance self-efficacy and performance by focusing attention, supporting self-regulated learning, or fostering flow. Effective goal setting depends on the task, situational constraints, and the type of focus required, and should ensure self-concordance, that is, alignment with one’s values, and adaptability. Our thematic analysis of ten performing artists’ goal setting during a coaching intervention illuminated the value of written goals and ways of setting long-term goals and showed that participants were aiming to be natural, spontaneous, and present. Further research is essential to deepen our understanding of goal setting across artistic domains and its potential to foster thriving creative practices. While using nature as a metaphor for long-term goals may be anthropomorphic, the principles of adaptability and synergy in natural ecosystems offer valuable insights for understanding how to thrive and cultivate a sustainable, fulfilling artistic life.

## 1 Introduction

Goal setting is a fundamental concept in both practice and research across different domains such as sports, business, and education (Bird et al., 2024; Locke and Latham, 2019; Martins van Jaarsveld et al., 2025). Goals can have a positive effect on performance (Williamson et al., 2024a) through mechanisms such as focusing attention, supporting self-regulated learning, and fostering the experience of flow (Csikszentmihalyi et al., 2018; Locke and Latham, 2002; Zimmerman, 2000). Despite these advantages and the frequent use of goals in practice, goal setting in the performing arts has been investigated to a much lesser extent (Lubert, 2024; Lubert and Gröpel, 2025; Nordin-Bates, 2023). Against the backdrop of the question of what drives individuals to thrive in the performing arts, we present an overview of various approaches to goal setting, drawing on recent reviews from sport and health psychology and insights from a qualitative case study with performing artists. We will look at goal setting through a holistic lens: by seeking inspiration in nature as a metaphorical heuristic and exploring what we can learn and adapt from its synergistic processes and how they can help us thrive as both humans and performers of acting, dance, or music.

Previous research on goal setting in the performing arts has focused on musicians and showed promising results. Interventions using goal setting seemed to enhance musicians’ sense of competence and self-efficacy (Hatfield, 2017; Lubert and Gröpel, 2022). Effective goal setting may not only improve the efficiency of music practice but also free up time for a healthier lifestyle (Taylor, 2021). Intrinsic goals—such as enjoying the music, having fun, or aiming for a rewarding performance experience—have been shown to contribute both to better musical outcomes and to greater life satisfaction (Lacaille et al., 2005, 2007). Intrinsic goals may thus be a meaningful way to set goals as a performing artist.

Another approach is based on the self-concordance model, which suggests that autonomous goals aligning with one’s personal values can significantly increase well-being (Sheldon, 2014; Sheldon et al., 2019; Sezer et al., 2024). Research shows that while individuals often do not know what to aim for to best promote well-being or personality development, self-concordant goals still balance a lack of anticipated need satisfaction and yield important benefits (Werner and Milyavskaya, 2018). Therefore, performing artists may be most likely to thrive if they can identify such self-concordant goals and commit to those that align with their natural talents and implicit processes (Sheldon, 2014; Sheldon et al., 2019).

## 2 Definitions of success in nature and the performing arts

Goal setting is inextricably linked to definitions of success, which sometimes appear to be taken for granted in high-performance domains. From the perspective of sport psychology, performing artists may appear “modest in their goal setting,” aiming, for example, to survive or to make a living, whereas athletes usually strive for the Olympic Games (G. Kenttä, personal communication, September 28, 2022). Looking at musicians’ success from a data-driven perspective, Kang et al. (2022) almost casually state that “usually, success is defined as being outstanding in the field” (p. 6). They operationalized success using Spotify popularity scores, follower counts, and appearances on the Billboard’s Hot 100, analyzing these in relation to the musicians’ collaboration networks and career spans. Importantly, classical composers ranked highest in terms of network influence—despite the seeming mismatch with the platform’s mainstream usage. Metrics-driven conceptions of success tend to neglect essential qualities like collaboration, resilience, and long-term development, which are qualities that sustain complex systems in nature (Folke et al., 2004). Furthermore, reliance on algorithmic metrics makes it difficult for aspiring musicians to succeed, limiting the control they can exert over their career paths (Musgrave, 2025). It is therefore worth questioning whether metrics are truly the sole means of demonstrating artistic success, or whether alternative criteria might better capture its essence.

In a qualitative study with focus groups on success and wellbeing, music students’ definitions included their relationships and social or daily environment, development, happiness, meaningfulness, balance, and authenticity as core aspects of both success and wellbeing (Rose et al., 2021). Health, safety, vitality, and attitude were unique to wellbeing, whereas recognition, career, financial goods, and achieving objectives—for example, reaching one’s goals—were unique to success. The students also thought that wellbeing and success influenced each other, whereby wellbeing was necessary, but not sufficient, for success. While these findings draw a multifaceted image of what success may mean for aspiring professional artists, they also emphasize the individual nature of such meaning and should be seen in the context of tertiary music education. Future research should thus illuminate meanings of success across different career paths, genres, and performing arts domains.

In nature, resilience and growth unfold in synergetic ecosystems. Success, that is, survival and thriving, do not depend on isolated strength but on relationships—nutrient cycles, mutualism, diversity, and feedback loops (Elmqvist et al., 2003). An organism thrives not just through its own capacity but by being embedded in a dynamic, responsive system. Similarly, when taking nature as inspiration, artistic success could be seen less as individual fame and more as being meaningfully connected: co-creating, connecting with others, and sustaining artistic vitality over time—thriving not through control, but through balance, rhythm, and adaptability. In ecological systems, species coexist, resources are shared and cycled, and change is constant (Jentsch and White, 2019). Growth happens in pulses, not linearly or toward fixed endpoints—honoring both effort and recovery, both structure and improvisation (Arnoldi et al., 2018; Han et al., 2025). Applying these ideas to the performing arts might shift the goals from being fixed and extrinsic to becoming more process-focused, aligned with inner values, and sustainable over time.

We do not claim to provide a definitive answer to how artistic success should be defined; rather, we invite readers to reflect on this question and on what nature, as a metaphorical heuristic, might offer in this regard. From a systemic perspective, it becomes evident that the focus cannot rest solely on the individual artist; the environment and conditions of artistic work must also be considered—and, where necessary, be transformed.

## 3 Expanding theoretical approaches to goal setting

Previous research has provided extensive evidence for the effectiveness of SMART goal setting: Specific, Measurable, Achievable, Realistic, and Time-bound (Doran, 1981). As a simple and memorable heuristic, SMART is appealing, seems inherently practical, and has been widely used (Swann et al., 2023). Despite the popularity of goal-setting theory by Locke and Latham (1990, 2019), the SMART heuristic was not derived from this or any other theory, and has recently been criticized not only for its lack of theoretical base, but also for a lack of empirical evidence, consideration of goal type, and consistent application (Swann et al., 2023, Schweickle et al., 2017). In an intervention study with musicians, the first author found that SMART goal setting did not improve self-efficacy compared to pre-performance routines (Tief and Gröpel, 2021). In a second study, she additionally encouraged participants to use different goal types (Lubert and Gröpel, 2022). Results showed that both goal-setting and pre-performance routines increased the self-efficacy of highly anxious participants. In both studies, the participants rated the interventions as helpful, but during one-on-one instructional sessions, the first author observed setting goals *per se* was helpful and not necessarily dependent upon the SMART heuristic.

Goal-setting theory states that under certain circumstances, specific, challenging goals are better than vague, “do-your-best” goals (Locke and Latham, 2002). When learning a new skill or being confronted with a difficult task, however, do-your-best goals may be more beneficial. Recent research in sport and health psychology highlights the value of such non-specific goals that “have some ambiguity or diffuseness in the exact levels of performance required” (Wallace and Etkin, 2018, p. 1034; Swann et al., 2025). Among these are not just do-your-best goals aiming try one’s best in general, but also as-well-as-possible goals (AWAP), that is, doing as much as one can for a specific task (Wallace and Etkin, 2018; Williamson et al., 2024b). This is a subtle but important difference: an AWAP goal is implicitly open-ended but tied to task constraints. It encourages an individual to maximize performance within a given context. AWAP goals are flexible and adaptive, making them especially useful in contexts where performance is hard to quantify or where conditions may change—such as in creative or high-pressure environments. They help maintain motivation without the pressure of hitting a precise benchmark (Wallace and Etkin, 2018). Another approach is setting range goals with two reference points that define the span within which one aims to perform (Scott and Nowlis, 2013; Wallace and Etkin, 2018). Lastly, non-specific goals can be open goals—more exploratory, curiosity-driven intentions such as “Let’s see how well I can do today” (Swann et al., 2017, 2025).

The term open goal was first coined for goals elite athletes described in relation to their experiences of optimal psychological states, such as flow (Swann et al., 2016, 2025). Open goals foster motivation, reduce pressure, and can be especially helpful when facing uncertainty, plateaus, or creative blocks. They allow for adaptability—mirroring natural systems that adjust flexibly to change. Flow describes an optimal state of awareness and consciousness, in which the task matches one’s level of skill mastery and that allows to perform without effort (Aherne et al., 2011; Csikszentmihalyi, 2008; Jackson and Kimiecik, 2008). There are indications that reaching a state of flow during performing is a sign of a performance-enhancing state (de Manzano et al., 2010; Harmat et al., 2021; Harris et al., 2023).

## 4 Empirical perspective

In a mixed-methods case study of interventions for managing performance anxiety with ten musicians, dancers and actors, goal setting was used to support coping with the challenges of performing (Lubert et al., 2023, 2025). Qualitative and quantitative analyses of data indicated positive effects of tailored individual coaching sessions on performance anxiety, fear of negative evaluation, self-efficacy, self-confidence, and expert-rated performance quality (Lubert et al., 2023). For the present paper, the recordings of the 50 coaching sessions (5 per participant) were re-analyzed using reflexive thematic analysis (Braun and Clarke, 2022), following the research question of how performing artists set and perceive their goals during a coaching intervention for performance anxiety management. Four themes were developed: (1) The value of written goals, (2) Aiming to be natural and spontaneous, (3) Aiming to be present, and (4) Setting long-term goals for being an artist.

The first theme comprised benefits of written goals. Jazz trumpeter Tom^1^ reflected back on the beginning of his coaching process: “I already found it helpful to simply write down certain goal ideas… it makes the whole thing somehow rationally tangible, in which direction it’s supposed to go.” Jazz singer Zoe elaborated on how written goals guided her both at the beginning and through a stressful time: “When we set the goals first. … I was very sure about the points that I gave you, so I was already like: ‘then why don’t I do it?’ Like ‘I can do it. I can achieve it.’ And I did actually and having a plan for the last 3 weeks—oh my God! It helped a lot. I also wrote it on computer and printed it and put it on my wall and I could see everything I should do. And I was like: ‘yes! Did that, did that, did that,’ and it worked. It really worked, and I didn’t feel that chaotic during those weeks because I could see.”

The second theme may be in contrast to the structure of specific written goals yet connected to thinking in natural processes: participants described aiming to be natural and spontaneous in their artistic work, referring to being able to improvise, to perform trusting and relying on their artistic abilities. For example, actor Lucy wanted “that there somehow is no more clear division between what is acting and what is reality. That the texts flow naturally, the situations naturally appear as if they actually emerged in the moment.” And dancer Coco said: “I’m looking for this spontaneous ability. To be able to do things on the spot. And be so solid as myself, as a dancer, even if/when even if I have flaws, that I can, you know, know my strengths, and know my flaws, and then play with that spontaneity.” These are examples of exploratory, open goals that can facilitate the experience of flow. Another aspect of this theme was nature-inspired metaphors, such as the intention to imagine a calm river as part of a pre-performance routine, or the goal of daring to jump across a river to describe taking a spontaneous risk in the moment when being on stage.

The third theme depicts how participants aimed to be in the present moment. This was particularly relevant for managing stress, as Mia, a baroque violinist who had just accepted several projects as a concertmaster, stated: “The most important point of preparation for me is probably to reach some kind of mindset and calmness. So everyone can have that, and everyone can feel safe and work in a safe environment, which I am—so the conductor and me—we are responsible for. I think that’s the biggest goal of mine for this whole crazy half a year ahead: to somehow maintain … this slow thinking … The main point of it is that I’m not in the past and I’m not in the future but I’m here.” Aiming to be present also alleviated pressure, as explained by Lucy: “Then it just was my goal to say ‘I now want to immerse myself in the moment, be completely in it. And everything else doesn’t matter, if I miss some notes, doesn’t matter, if the text gets messed up, doesn’t matter. But you are in the role now, in the moment.’ That was my goal.” Being in the present moment not only can be conducive for a beneficial attentional focus under pressure but also to optimal psychological experiences, where research has shown that experiencing a state of flow is incompatible with the experience of debilitating performance anxiety (Cohen and Bodner, 2021; Fullagar et al., 2013; Harmat et al., 2021).

The fourth theme captured a more long-term perspective on goals for being an artist. Participants alluded to their motivation to connect with audiences, as described by cellist Eva: “When I attend like really good concerts, then I just keep having a certain mood the whole evening or also the day after … that’s fascinating indeed. … so this is somehow a bit of my goal, that I [as a musician] can affect people like that, too.” Similarly, accordionist Julia said: “That’s somehow my goal: music as a whole.” In another session, she expressed her long-term aspirations in a slightly different nuance: “This would be such a goal for me … to really be able to enjoy that I am allowed to be a musician.” Thus, the long-term goals expressed by participants were intrinsic and inspired by sustainable ideas of success. They focused on meaningful relationships with others and a healthy relationship with oneself and one’s artistic craft.

## 5 Discussion

The four themes indicate that participants acknowledged different goal types—written, unwritten, specific and non-specific, short- and long-term—as important for being a performing artist. Thinking back to how natural systems thrive, actors, dancers, and musicians may need a more dynamic system of goals and resources to succeed than the one suggested by goal-setting theory and the SMART framework. Success, as a goal itself, could be viewed through a more ecological and nature-aligned lens rather than being defined solely by individually focused metrics such as album sales, ticket revenue, or social media followers.

To our knowledge, previous research in music has only investigated written goals, and apart from the presented case study, there are no empirical studies on goal setting in dance and acting (Lubert, 2024; Nordin-Bates, 2023). In dance psychology, principles of goal setting from sport, exercise, and organizational psychology have been convincingly transferred to dance contexts, with the most important distinction being that dance is a subjective art form (Nordin-Bates, 2023). Nordin-Bates’s recommendation to make subjective, artistic goals as specific as possible may indeed serve to avoid vagueness and increase clarity for each individual artist, not just in dance but also in music, and perhaps especially in acting, which appears to have the fewest objective parameters of the three domains (e.g., no measurable tempo). It may also be important to consider that goals in music and dance are often set by teachers in classes and lessons, whereas actors may be more independent, at least in their initial preparation process. Nonetheless, we suggest that all performing artists would equally benefit from a better understanding of goal setting—a claim that still requires further investigation in future research studies.

Different goal types serve different functions, depending on situational demands and contextual factors: when goals are inherently time-bound, it can be helpful to write down everything that needs to be done to keep an overview and reduce stress (Burwell and Shipton, 2013). Furthermore, performance profiling—inherently providing written goals—shows promising results for musicians (Hatfield, 2024). However, a study with athletes comparing no goals, unwritten goals, written goals, or written and displayed goals showed no differences in performance, commitment, motivation, or perceived goal difficulty (Weinberg et al., 2019). And even though looking at one’s goals increased the likelihood of individual practice, this did not lead to enhanced performance. Results may have been influenced by control participants not being specifically instructed to set goals, whereas for the other three groups, goals were set by the researchers. Coaching studies indicate that goals and tasks are more effective if initiated by the coachee rather than the coach (Gessnitzer and Kauffeld, 2015; Lubert et al., 2025). In practice, it may be individual and dependent on the situation whether writing down goals is perceived as helpful or not.

There is little research on non-specific goals in sport (Williamson et al., 2024a,b), and none in the performing arts. To address this gap, further research is needed to examine the context in which different types of goals are most effective across artistic domains. In music and dance, performance goals can be especially maladaptive because musicians and dancers are particularly vulnerable to performance pressures, perfectionism, and anxiety (Nordin-Bates, 2023). Mastery goals, however, have been associated with adaptive dispositions and favorable outcomes (for a review, see Tan and Sin, 2020). The previous results of the case study suggested that participants were at least partly able to reach their goals, but some were especially mindful of being in a long-term developmental process (Lubert et al., 2023, 2025). Therefore, it is important to recognize that connection, artistic joy, and mastery represent meaningful and sustainable aims when considering beneficial long-term goals, which can be closely linked to the concept of success.

The empirical perspective of this paper is limited by its post-hoc research question focused on goal setting and the secondary, briefly summarized analysis. Another limitation is the metaphor of nature in the context of long-term goals, which may arguably be an anthropomorphic perspective. Although engaging in creative activities such as music-making has well-documented benefits for well-being and mental health, performing artists remain particularly susceptible to a range of health challenges, including depression and (performance) anxiety (Détári et al., 2020; MacDonald, 2013; Musgrave, 2022). Similar to natural systems, performing artists often operate within demanding and rapidly changing work environments (Détári et al., 2020). Thriving ecosystems must constantly adapt to shifting conditions in order to survive and thrive. Investigating the mechanisms that enable such resilience can provide valuable insights. Drawing parallels with natural cycles of productivity and periodization may be especially fruitful (Manchester, 2008; Wyon and Allard, 2021). Looking to nature for inspiration may foster future research on thriving in the performing arts, not only with relation to goal setting but also time management, health and personal development.

To conclude, drawing inspiration from nature as a metaphorical heuristic may not only offer valuable insights into the meaning of success but also help set goals in a sustainable way. In this paper, we aim to contribute to the psychology of the performing arts by combining three elements: (a) extending established approaches to goal setting through alternative goal types, (b) integrating qualitative insights into performing artists’ goal-setting practices, and (c) incorporating perspectives inspired by natural processes. This synthesis provides a more holistic framework for understanding and supporting goal setting in demanding, dynamic artistic environments. On a practical level, effective goal setting for actors, dancers, and musicians depends on the task, situational constraints, and the required focus. It may involve SMART goals, do-your-best or as-best-as-possible goals, or open, exploratory goals—always with an emphasis on self-concordance, that is, being in tune with one’s inner values, and being able to adapt.

## Data Availability

The data analyzed in this study is subject to the following licenses/restrictions: The datasets analyzed for this paper are available on request to the corresponding author. Requests to access these datasets should be directed to VL, veronika.lubert@hslu.ch.

## Footnotes

1 All names are pseudonyms.
